# Polyphenols in Colorectal Cancer: Current State of Knowledge including Clinical Trials and Molecular Mechanism of Action

**DOI:** 10.1155/2018/4154185

**Published:** 2018-01-15

**Authors:** Md Nur Alam, Muhammad Almoyad, Fazlul Huq

**Affiliations:** Discipline of Biomedical Sciences, Sydney Medical School, The University of Sydney, Cumberland Campus C42, East Street, Lidcombe, NSW 1825, Australia

## Abstract

Polyphenols have been reported to have wide spectrum of biological activities including major impact on initiation, promotion, and progression of cancer by modulating different signalling pathways. Colorectal cancer is the second most major cause of mortality and morbidity among females and the third among males. The objective of this review is to describe the activity of a variety of polyphenols in colorectal cancer in clinical trials, preclinical studies, and primary research. The molecular mechanisms of major polyphenols related to their beneficial effects on colorectal cancer are also addressed. Synthetic modifications and other future directions towards exploiting of natural polyphenols against colorectal cancer are discussed in the last section.

## 1. Introduction

Epidemiological studies exhibiting protective effect of diets rich in fruits and vegetables against different types of cancer have drawn increased attention to the possibility of exploiting biologically active secondary metabolites of plants to fight against cancer. Among the vast array of phytochemicals, compounds called “polyphenols” constitute one of the most numerous and widely distributed groups, covering more than 10,000 different chemical structures [1]. Polyphenols (PP) are reported to have antioxidant, anticarcinogenic, antiatherosclerotic, anti-inflammatory, spasmolytic, hepatoprotective, antiviral, antiallergic, antidiarrheal, antimicrobial, and oestrogenic activity [2].

Colorectal cancer (CRC) is the third most common diagnosed cancer in men after lung and prostate cancer throughout the world. While in women CRC occupies the second position after breast cancer worldwide. Prevalence of CRC is 18% higher in developed countries than developing and undeveloped nations. People of more than 50 years old are more prone to be affected by CRC, and incidence in males is greater than in females. Although diet and Western lifestyle are still considered as being the main factors responsible for CRC, no specific food or other environmental agent has been identified as an exact causative factor [3]. Thus far, clearly identified types or causes of CRC are hereditary nonpolyposis colorectal cancer, familial adenomatous polyposis, inflammatory bowel diseases, human papillomavirus, and acquired immunodeficiency syndrome [4]. Although surgical resection remains the only curative treatment for CRC, an alternative approach to reduce the mortality rate is chemoprevention, use of synthetic or natural compounds in pharmacologic doses [5].

Colon cancers result from a series of pathologic changes that transform normal colonic epithelium into invasive carcinoma. Dietary PP affect these different cellular processes by acting as chemopreventive blockers. So far, only one review article that has been published concentrated on the effect of polyphenols on colorectal cell lines [6], and only a limited number of polyphenols have been considered. This review focuses on the updated research on a wider variety of polyphenols as applied to colorectal cancer.

## 2. Chemistry of PP and Their Dietary Sources

**Figure 1:**
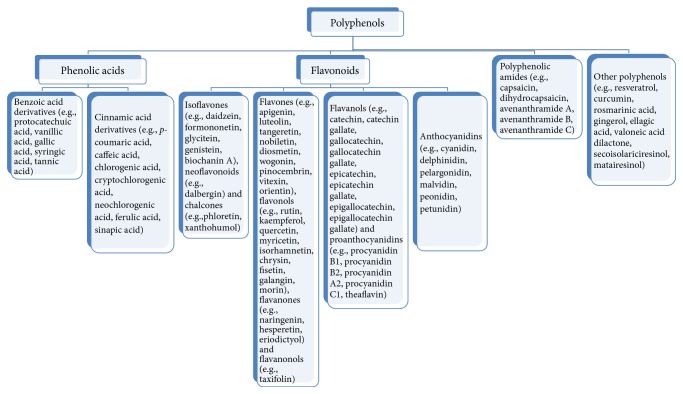
Classification of Polyphenols.

**Figure 2:**
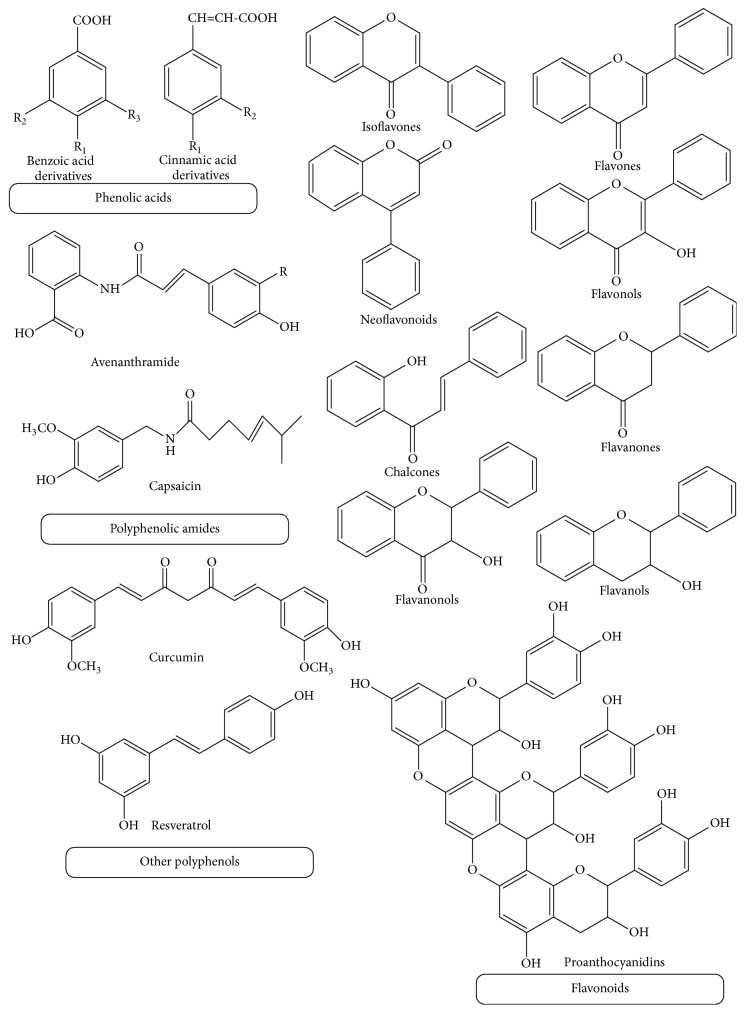
Basic structures of major groups of polyphenols.

PP are also known as polyhydroxyphenols and characterized by the presence of large number of phenol units in their structures, usually existing in plants as glycosides. Polyphenols can be classified according their sources, chemical structures, therapeutic actions, and so on. A classification system of PP has been given in Figure 1 on the basis of the chemical structures of the aglycone portions and Figure 2 gives the basic structures of major groups [7].

A list of the 100 richest dietary sources of PP has been produced using comprehensive Phenol-Explorer data [8]. The richest sources are various spices and dried herbs, cocoa products, some dark coloured berries, some seeds (flaxseed) and nuts (chestnut, hazelnut), and some vegetables, including olive and globe artichoke heads. Top ten of the list containing the highest amount of PP is in the following order: cloves > peppermint (dried) > star anise > cocoa powder > Mexican oregano (dried) > celery seed > black chokeberry > dark chocolate > flaxseed meal > black elderberry.

## 3. Pathogenesis of CRC and Its Signalling Pathways

Acquired functional capabilities of cancer cells that would allow them to survive, proliferate, and disseminate are known as the hallmarks of cancer, that is, sustaining proliferative signalling, evading growth suppressors, resisting cell death, enabling replicative immortality, inducing angiogenesis, activating invasion and metastasis, reprogramming of energy metabolism, and evading immune destruction [9]. Underpinning these hallmarks are genomic instability and inflammation. While genomic instability confers random mutations including chromosomal rearrangements, causing genetic diversity that expedites the acquisition of hallmarks of cancer, the inflammatory state of premalignant and frankly malignant lesions that is driven by cells of the immune system also fosters multiple hallmark functions.

Based on investigation of different stages of tumour initiation and progression, Fearon and Vogelstein proposed a model of colorectal carcinogenesis that correlated specific genetic events with evolving tissue morphology [10]. The Wnt/*β*-catenin pathway plays a dominant role in an initial stage of CRC development. Inactivation of the adenomatous polyposis coli gene is a key starting event in carcinogenesis of more than 60% of colorectal adenomas and carcinomas leading to stimulation of the Wnt pathway via free *β*-catenin [10].

Stimulation of the epidermal growth factor receptor (EGFR) leads to the activation of KRAS or phosphatidylinositol-3-kinase pathways, which is important in CRC development from early adenoma to intermediate adenoma. Subsequently, numerous signal transduction molecules initiate a cascade of downstream effectors that trigger tumour growth, angiogenesis, and metastasis [11].

Transforming growth factor-*β* (TGF-*β*) is a multifunctional polypeptide that binds to specific TGF-*β* receptors for paracrine and autocrine signalling. This ligand and receptor complex triggers intracellular signalling cascades that include the canonical Smad2 signalling pathway, which complexes with Smad4 and accumulates and translocates into the nucleus. In the nucleus, activated Smad complexes regulate the transcription of specific genes and ultimately regulate cell cycle and tissue repair [12]. TGF*β* pathway contributes to a favourable microenvironment for tumour growth and metastasis throughout all the steps of carcinogenesis [13]. TGF-*β* also induces apoptosis, from the association of death-associated protein 6 (DAXX) with the death receptor Fas. After binding, DAXX is then phosphorylated by homeodomain-interacting protein kinase 2 (HIPK2), which then activates apoptosis signal-inducing kinase 1 (ASK1). ASK1 activates the Jun amino-terminal kinase (JNK) pathway that causes apoptosis [14–16]. Inactivation of TGF-beta pathway components is first detected in advanced adenomas and affects 40–50% of all CRCs [17].

Almost 50% of all CRCs show p53 gene mutations, with higher frequencies observed in distal colon and rectal tumours and lower frequencies in proximal tumours and those with the microsatellite instability or methylator phenotypes [18]. The mutations in p53 or the loss of its functionality occurs mainly at the transition from adenoma to cancer, and the frequency of alterations in the gene increases with the corresponding progression of the lesion [19].

CRC cells share many properties in common with stem cells which are conserved in both dormant and actively proliferating cancer cells [20]. On top of maintaining “stemness” characteristics, CRC cells with metastatic potential dissociate from the tumour mass and spread to other organs in the body [21]. This is achieved through a dedifferentiation program called epithelial-mesenchymal transition (EMT). This key developmental program allows stationary and polarized epithelial cells to undergo multiple biochemical changes that enable them to disrupt cell-cell adherence, lose apical-basal polarity, dramatically remodel the cytoskeleton, and acquire mesenchymal characteristics such as enhanced migratory capacity, invasiveness, and elevated resistance to apoptosis [22]. Adhesion molecules that maintain cell-cell contact in the differentiated tumour cells, such as E-cadherin, are downregulated in the undifferentiated cells, while molecules that impart invasive and migratory behaviour would be upregulated. To accommodate both the “stemness” and mesenchymal properties of invasive CRC cells, it has been proposed that CRC cells with metastatic potential are like “migratory stem cells” [23]. The EMT process is initially driven by three core groups of transcriptional regulators described as follows. The first is a group of transcription factors (TFs) of the Snail zinc-finger family, including SNAI1 and SNAI2 (SLUG) [24]. The second group is the distantly related zinc-finger E-box-binding homeobox family of proteins ZEB1 and ZEB2 (SIP1) [25]. The third group is the basic helix-loop-helix (bHLH) family of transcription factors, including TWIST1, TWIST2, and E12/E47 [26]. In CRC, 85% of resected specimens have moderate to strong TWIST1 expression [27]. The earlier steps of the metastatic cascade EMT program include local invasion, intravasation, survival while transiting through the circulation, and extravasation. EMT programs are dynamically regulated, and during the last step of the metastatic cascade, colonization, carcinoma cells are thought to switch back to an epithelial state through the reverse process, mesenchymal-epithelial transition (MET) [28]. The final stage of the invasion-metastasis cascade, colonization, is likely to require adaptation of propagated CRC cells to the microenvironment of a distant tissue [29].

Increased matrix metallopeptidases (MMPs) expression and their activation generally promote hallmarks of CRC progression including angiogenesis, invasion, and metastasis and correlate with shortened survival. MMPs comprise a large family of at least 25 zinc-dependent endopeptidases capable of degrading all components of the extracellular matrix (ECM) and are categorized primarily by their structural features as gelatinases, collagenases, membrane-type, stromelysins, and matrilysins [30]. Intercellular adhesion molecule-1 (ICAM-1) is a 90-kDa cell surface glycoprotein that is known to be a member of the immunoglobulin gene superfamily of adhesion molecules. ICAM-1 expression is closely associated with metastasis and may be a useful indicator of prognosis in patients with colorectal cancer [31].

**Figure 3:**
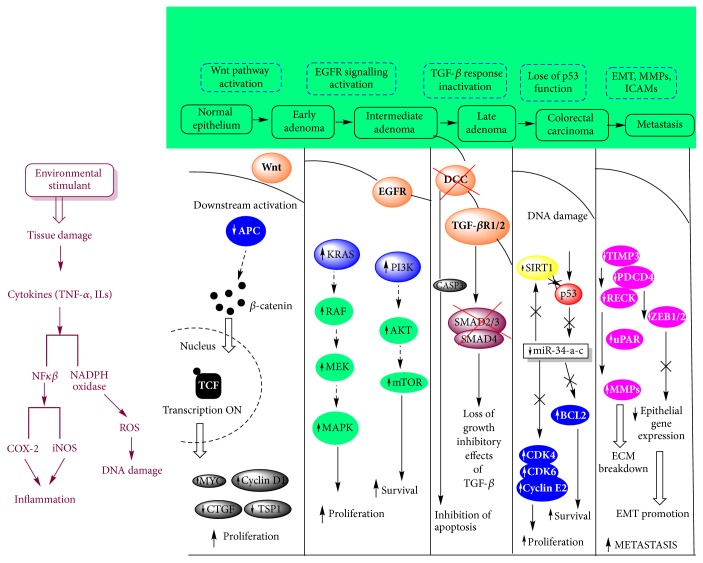
*Signalling pathways in colorectal cancer pathogenesis (adapted from [168])*. (EGFR: epidermal growth factor receptor, TGF*β* R1/2: transforming growth factor, beta receptor 1/2, EMT: epithelial-mesenchymal transition, ICAMs: intercellular adhesive molecules, MMPs: matrix metallopeptidases).

It is evident from the above discussion that the pathogenesis of CRC is characterized by regulatory pathways that are complex involving several layers of communication, cascades, crosstalk, and extensive networking. CRC usually develops through interaction of cytokines, the chemical mediators of inflammation; cytokine receptors, present on the surface of a variety of cell types; secondary messengers which convey signals from cell surface to the interior; transcription factors, which regulate the expression of several genes that affect CRC.  Figure 3 depicts the signalling pathways involved in CRC.

## 4. Roles of PP in CRC Related to Chemoprevention and Apoptosis

Consumption of PP rich food proved to be beneficial in occurrence of CRC in a national prospective cohort study [32]. Numerous studies have evaluated the efficacy of dietary polyphenols against CRC* in vivo*,* in vitro* model and in clinical trials [33–35]. Polyphenols can affect the overall process of carcinogenesis by several mechanisms and cause tumour cell death through apoptotic pathway.

PP have been shown to be highly effective in scavenging singlet oxygen and various free radicals, which leads to DNA damage and tumour promotion [36]. PP also displayed chemopreventive effect through their impact on the bioactivation of carcinogens. Most carcinogens of chemical origin undergo biotransformation by Phase I metabolizing enzymes to be converted into more reactive form suitable for binding with DNA and proceed towards carcinogenesis process. PP were found to inhibit cytochrome P450 enzymes of the CYP1A family and thus act as chemopreventive agents [37]. On the other hand, by increasing the activity of Phase II metabolizing enzymes (glutathione reductase, glutathione peroxidase, glutathione* S*-reductase, catalase, and quinone reductase), PP are able to provide beneficial effects against CRC [38, 39]. For example, PP obtained from apple inhibited growth of HT-29 human colon cancer cells by modulating expression of genes (*GSTP1*,* GSSTT2*,* MGST2*,* CYCP4F3*,* CHST5*,* CHST6*, and* CHST7*) involved in the biotransformation of xenobiotics [40].

Orner et al. demonstrated that epigallocatechin-3-gallate (EGCG) attenuated the expression of *β*-catenin and inhibited intermediate and late stages of colon cancer, via effects on the Wnt/*β*-catenin/TCF signalling pathway [41]. EGFR signalling mechanism of CRC progression has been reported to be inhibited by apple procyanidins [42]. Expression of p53 gene has been increased by EGCG that can impede the conversion of colorectal adenoma to colorectal carcinoma during carcinogenesis [43].

Apoptosis is a vital physiological process in the normal development, and induction of apoptosis is highly anticipated mode as a therapeutic strategy for cancer control [44–46]. Bcl family of protein, caspase signalling proteins, and p53 genes are the key factors that regulate apoptosis [47]. PP are effective general inhibitor of cancer cell growth and inducers of apoptosis in different cancer cell lines, including leukaemia, skin, lung, stomach, colorectal, and prostate cancer cells [34, 48–52]. Anthocyanin, ellagic acid, curcumin, flavone induced apoptosis in various colon cancer cell lines by different mechanisms in miscellaneous observations [34, 53–55].

PP can prevent the DNA damage caused by free radicals or carcinogenic agents through diverse mechanisms: (a) direct radical scavenging [56, 57], (b) chelating divalent cations involved in Fenton reaction [58], and (c) modulation of enzymes related to oxidative stress (glutathione peroxidase, glutathione reductase, superoxide dismutase, nitric oxide synthase, lipooxygenase, xanthine oxidase, etc.) [59]. Dietary PP can also act as prooxidants depending on the cell type, dose, and/or time of treatment, as they can enhance reactive oxygen species production and therefore induce apoptosis [58, 60, 61]. In colon cancer HT-29 cells, flavone enriched the mitochondrial pyruvate or lactate uptake, which augmented the superoxide radical production and led to apoptosis [62].

## 5. Recent Update of Key PP as Applied to CRC

Reported antitumour activity of PP against CRC is largely based on* in vitro* studies, rodent model studies, and even human clinical trials. During* in vitro* studies on antitumour activity of PP, different colorectal cancer cells (HT-29, SW480, Caco-2, Colo-205, Colo-115, HCT-115, HCT-116, DLD-1, LoVo etc.) were cultured, and cell viability was determined via MTT [3-(4,5-Dimethylthiazol-2-yl)-2,5-Diphenyltetrazolium Bromide] reduction assay [154], SRB (Sulforhodamine B) colorimetric assay [155], and crystal violet method [156].* In vivo* animal model models were produced by inducing tumour chemically, resulting in APC^Min/+^ mouse and rodent xenograft models. Carcinogen [azoxymethane (AOM), dimethylhydrazine (DMH), dextran sodium sulphate (DSS)] induced colon cancer in rodents can recapitulate in a highly reliable way and frequently used to assess activity of PP. Mutations in the adenomatous polyposis coli (APC) gene are required to initiate familial adenomatous polyposis (FAP) and are also important in CRC tumorigenesis. Several studies have been conducted with PP in APC^Min/+^ mouse that contains (multiple intestinal neoplasia* Min*) a point mutation in the* APC* gene and develops numerous adenomas. The role of PP has also been investigated in xenograft model where human tumours are injected and established in immunodeficient mouse strains (nude or SCID mice). This section contains the outcome from the studies conducted with PP against CRC.

### 5.1. Phenolic Acids

#### 5.1.1. Benzoic Acid Derivatives

From literature, only gallic acid among benzoic acid derivatives showed anticancer activity against CRC* in vitro* and* in vivo* model [157, 158], but no study has been conducted to identify the anticancer mechanism of gallic acid in CRC. However, gallic acid is believed to exhibit its anticancer effect by upregulating Bax and downregulating Bcl-2 in other tumour models [157]. Vanillic acid showed significant activity with IC_50_ values less than 30 *μ*M in three different CRC cell lines but mechanism has not been studied [159] although vanillic acid and protocatechuic acid did not show significant anticancer activity against CRC [160, 161].

#### 5.1.2. Cinnamic Acid Derivatives

Caffeic acid showed apoptotic cell death against HCT 15 cell lines although IC_50_ value was very high (800 *μ*M). Similar findings were made by other researchers [162, 163]. In a recent study caffeic acid did not show any significant activity against HT-29 cell lines up to 200 *μ*M concentration nor did chlorogenic acid [164] that did not show any significant activity against different human colorectal carcinomas [161]. IC_50_ values of* p*-coumaric acid against some other CRC cell lines were around 1 mM and apoptosis was the mechanism of cell death [163, 165, 166]. Ferulic acid inhibited CRC progression at adhesion and migration steps but no IC_50_ value was greater than 1 mM concentration [163].

Carnosic acid showed IC_50_ values in the range of 24–96 *μ*M against Caco-2, HT29, and LoVo cell lines. It inhibited cell adhesion and migration, possibly by reducing the activity of secreted proteases such as urokinase plasminogen activator and metalloproteinases. These effects may be mediated through a mechanism involving the inhibition of the COX-2 pathway [167]. Sinapic acid showed IC_50_ values of less than 25 *μ*M in three different CRC cell lines but mechanism has not been studied [159].

### 5.2. Flavonoids

#### 5.2.1. Isoflavones, Neoflavonoids, and Chalcones

Among isoflavones, biochanin A showed ID_50_ values below 15 *μ*g/mL against two CRC cell lines and was found to enhance radiotoxicity* in vitro* [170, 171]. Formononetin that showed dose dependent cell killing, both* in vitro* and* in vivo* in RKO cell line, induces apoptosis by modulating Bax/Bcl-2 activities, inactivating ERK pathway and TNF-*α*/NF-*κ*B pathway [172]. Formononetin also showed anticarcinogenic activity in HCT-116 cells via promotion of caspase-dependent apoptosis and inhibition of cell growth, with contribution by downregulation of the antiapoptotic proteins Bcl-2 and Bcl-xL [173]. Daidzein killed 50% of HCT cells, LoVo cells, and DLD-1 cells at concentration of 40 *μ*M, 68.8 *μ*M, and 46.3 *μ*M, respectively, but against LoVo cells it exhibited biphasic effects by killing cells in dose dependent manner at higher concentrations (≥5 *μ*M) and vice versa at lower concentrations (≤1 *μ*M) [75, 174, 175]. Most commonly studied isoflavone, genistein, showed cytotoxicity against HCT, LoVo, and DLD-1 cell lines with IC_50_ values of 15 *μ*M, 57.3 *μ*M, and 56.1 *μ*M, respectively, whereas in HCC-44B2 cells and HCC50-D3 the value was 11.5 *μ*g/mL and 9.5 *μ*g/mL [75, 170, 174]. Genistein reduces the density of cell surface charge and increases the order in membrane protein conformation which might be one of the mechanisms of its anticancer effect [174].

 No literature reporting neoflavonoids activity against CRC was found. Among chalcones, phloretin caused apoptotic cell death to HT-29 cells with an IC_50_ value close to 100 *μ*M. The mechanism involved changes in mitochondrial membrane permeability and activation of the caspase pathways [176]. Phloretin also has the potential to increase adoptive cellular immunotherapy against SW-1116 CRC cells [177]. Xanthohumol, another important chalcone, is found to show cytotoxicity in different CRC cells* in vivo* and* in vitro* with IC_50_ values less than 5 *μ*M [178–180]. The apoptosis involved downregulation of Bcl-2, activation of the caspase cascade, and inhibition of topo I activity. In combination with chemotherapy, it is recommended for use in HCT-15 cell lines, being aimed to reduce drug resistance by inhibition of efflux transporters [180]. Xanthohumol inhibits metastasis by inhibiting expression of CXCR4 chemokine receptor [181].

#### 5.2.2. Flavones, Flavonols, Flavanones, and Flavanonols

Among all different types of flavones, apigenin and luteolin were most commonly investigated phytochemicals for their anticancer activity against CRC. Important flavones that have been studied against CRC are given in Table 1.

Among all different types of flavonols quercetin, chrysin and rutin were studied most for their anticancer activity against colorectal cancer models. Important flavonols that have been investigated against CRC are given in Table 2.

Naringenin appears to be the most commonly studied phytochemicals among the flavanones that can act against colorectal cancer. It suppressed colon carcinogenesis through the aberrant crypt stage in azoxymethane-treated rats [74]. Another study showed that antiproliferative activity of naringenin was estrogen receptor dependent [182], while other* in vitro* studies gave mixed results in different CRC cell lines [65, 80, 152, 183]. Another flavanone, hesperetin, significantly reduced the formation of preneoplastic lesions and effectively modulated the xenobiotic-metabolizing enzymes in rats during DMH-induced colon cancer study [184, 185].

Among flavanonols, only taxifolin acts as an effective chemopreventive agent against colon carcinogenesis due to its antioxidant mediated apoptosis and antiproliferative activities [186, 187]. Taxifolin is found to control NF-kB-mediated Wnt/*β*-catenin signalling via upregulating Nrf2 pathway [188].

#### 5.2.3. Flavanols and Proanthocyanidins

**Figure 4:**
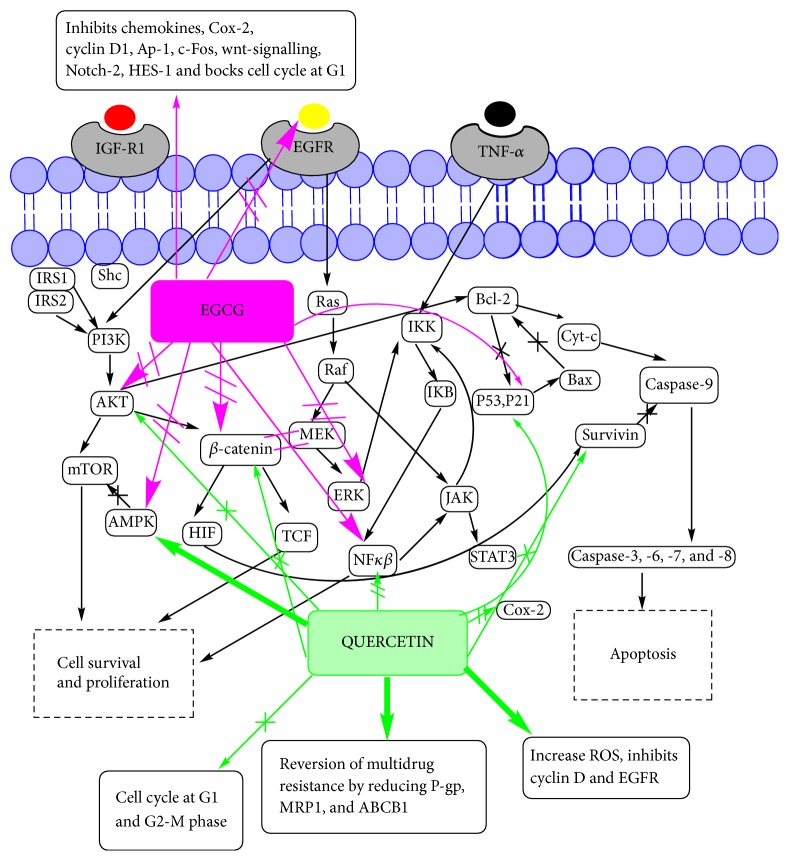
Molecular mechanism for anticancer action of EGCG and quercetin in CRC.

Epigallocatechin gallate (EGCG) is the most studied flavanols against CRC. EGCG showed IC_50_ values of 42.2 *μ*M, 47.7 *μ*M, 50.2 *μ*M, 80.1 *μ*M, and 43.1 *μ*M against HCT116, HT29, SW480, and SW837 cell lines, respectively. Mechanism of action has been linked to the inhibition of growth and activation of the epidermal growth factor receptor and human epidermal growth factor receptor-2 signalling pathways [189]. Among eleven different types of flavanol, EGCG showed the highest antiproliferative activity against HCT-116 cells at 50 *μ*M [190]. In APC^Min/+^ mice, EGCG significantly inhibited intestinal tumorigenesis. Oral administration of EGCG showed increased levels of E-cadherin and decreased levels of nuclear *β*-catenin, c-Myc, phospho-AKT, and phospho-extracellular signal-regulated kinase 1/2 (ERK1/2) in small intestinal tumours [191]. In another mice model, EGCG reduced inflammation-related colon carcinogenesis induced by azoxymethane and dextran sodium sulphate. Antitumour activity has been ascribed to decrease in mRNA expression levels of COX-2 and inflammatory cytokines (*TNF-α*,* IFN-γ*,* IL-6*,* IL-12*, and* IL-18*) in the colonic mucosa [192]. Other studies mentioned the proposed anticancer mechanisms of EGCG including cell cycle arrest and apoptosis through inhibition of cyclooxygenase-2 expression, activation of AMP-activated protein kinase, cyclin D1 degradation and p21 transcriptional activation, and inhibition of HES1 and Notch2 signalling in different colorectal cancer cell lines [43, 193–196]. EGCG also inhibited invasion and MMP expression, angiogenesis, through blocking the induction of VEGF [197, 198]. Molecular mechanism for antitumour activity of EGCG and quercetin is shown in Figure 4.

Chemopreventive effects of theaflavin have been reported in azoxymethane induced colon cancer study in male Sprague Dawley rats [199]. The mechanism behind the beneficial effects of proanthocyanidins against CRC has been related to inhibition of angiogenesis through suppressing the expression of VEGF and Ang1 [200], induction of apoptosis [201], and antioxidant activity [202].

#### 5.2.4. Anthocyanidins

Malvidin and pelargonidin showed IC_50_ values of 71.7 *μ*g/mL and 154.3 *μ*g/mL against HCT-116 cell line, whereas cyanidin, delphinidin, and petunidin did not induce 50% cell killing even at a concentration of 200 *μ*g/mL [203]. Cyanidin, delphinidin, malvidin, and pelargonidin exhibited no cytotoxicity against Caco-2 cell line, but against metastatic LoVo and LoVo/ADR cell line cyanidin and delphinidin showed significant antitumour activity with low IC_50_ values [204]. Anthocyanin or anthocyanidin containing extracts obtained from a variety of sources were reported to have significant antiproliferative activity against CRC in several animal model (APC^Min/+^ mouse model, chemically induced) and cell lines [205–213]. Anticancer activity of anthocyanidins is believed to be due to its antimetastatic property through modulation of claudins, matrix metalloproteinases (MMPs), nuclear factor *κ*B (NF-*κ*B) activation, and demethylation of tumour suppressor genes [214–216]. A pilot study involving 25 CRC patients showed 7% decrease in proliferation after consumption of anthocyanin rich diet [217].

### 5.3. Polyphenolic Amides

Capsaicin is the most studied polyphenolic amide against CRC.* In vitro* and* in vivo* studies in mice bearing Colo-205 tumour xenografts showed that capsaicin significantly reduced tumour progression by activating caspase-3, caspase-8, caspase-9, Bax, Fas and reducing Bcl-2 [218]. Capsaicin and 3,3′-Diindolylmethane worked synergistically against CRC via modulating transcriptional activity of NF-*κ*B, p53, and target genes linked with apoptosis [219]. Other studies showed that anticancer action of capsaicin was related to nitric oxide production, reactive oxygen species generation, and suppression of transcriptional activity of *β*-catenin/TCF pathway [220–222]. In our study, we have found synergism in binary sequenced combination of capsaicin with oxaliplatin in all sequences (0,0 h; 0,4 h; 4,0 h) and more so in higher concentration in Lim 2405 cell line (unpublished data). Dihydrocapsaicin, a saturated structural analogue of capsaicin, was found to possess greater activity than capsaicin against HCT-116 cells and induced autophagy in a catalase-regulated manner [223]. Avenanthramides significantly inhibited proliferation of HT29, Caco-2, LS174T, and HCT116 human colon cancer cells [224].

### 5.4. Other Polyphenols

#### 5.4.1. Resveratrol

**Figure 5:**
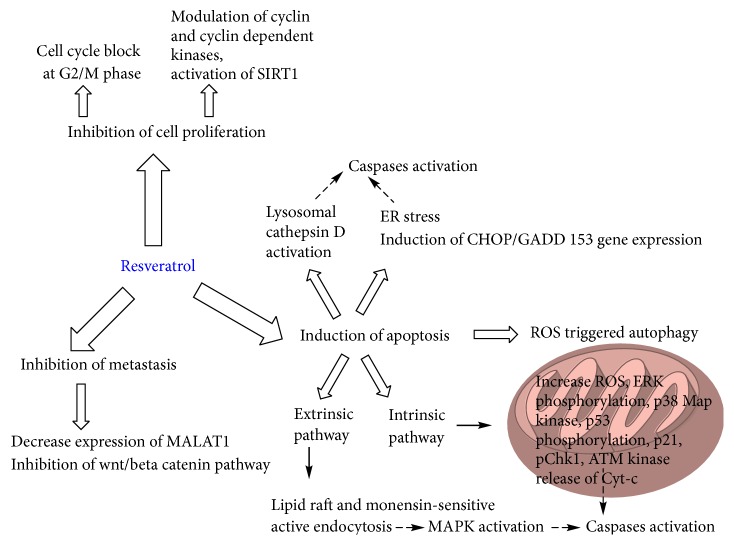
Molecular mechanism for anticancer action of resveratrol in CRC.

3,5,4-Trihydroxystilbene, known as resveratrol, is one of the most studied polyphenols against CRC. It entered into clinical trial after a number of preclinical studies for its encouraging activity and nontoxicity. All studies conducted in rodent model and clinical trial regarding the activity of resveratrol up to 2009 have been described by Bishayee in his review [225]. The authors mentioned 9* in vivo* studies related to CRC, among which three were conducted in APC^Min/+^ mice model and others related to chemically induced tumour [226–234]. All the studies discussed in the review (except one on APC^Min/+^ mice study) provided support for therapeutic potential of resveratrol against CRC. Later on, in 2012, Juan et al. published another review article that focused on the effects of resveratrol on CRC from conducted* in vivo* studies and clinical trials [235]. The authors provided details on the molecular mechanism of action of resveratrol against CRC. Two more research articles recently described the promising effect of resveratrol in mouse model against CRC, which were not mentioned in earlier reviews [236, 237]. The results from the investigations of the anticancer activity of resveratrol against CRC have not been detailed here because they have been considered well in the previously mentioned reviews. However, a pictorial representation of the molecular mechanism of action of resveratrol against CRC is given in Figure 5. The effect of 5-fluorouracil increased in combination with resveratrol due to chemosensitizing property [238]. Antimetastatic activity of resveratrol in CRC has also been reported [237, 239]. Since resveratrol is found to downregulate multidrug resistant protein 1 by preventing activation of NF-*κ*B signalling and suppressing cAMP-responsive element transcriptional activity, it can be used to overcome drug resistance by combining with other chemotherapeutic drugs [240]. We have found synergism at higher concentration in binary sequenced combination (at 0,0 h; 0,4 h; 4,0 h) of resveratrol with cisplatin but additive to antagonism at lower concentration in HT-29, Caco-2, and Lim 2405 cell lines (unpublished data).

#### 5.4.2. Curcumin

**Figure 6:**
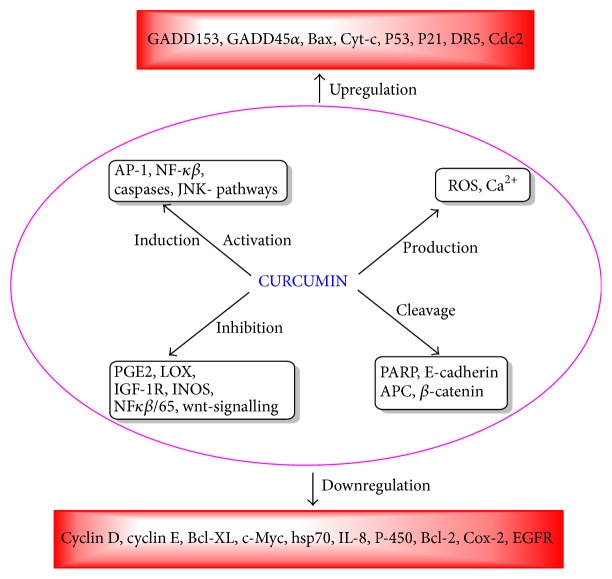
Different pathways involved in apoptosis by curcumin in CRC.

Curcumin is the main active compound in turmeric (dried rhizome of* Curcuma longa*). We have found 82 research articles (*in vitro* and* in vivo* preclinical studies, clinical trial) describing effect of curcumin including mechanism of action against CRC. Here, we have not considered the anticancer activity of curcumin in detail because three review articles discussed well the therapeutic potential of curcumin for CRC along with its mechanism of action [241–243]. Curcumin can inhibit the initiation of carcinogenesis by increasing glutathione S- transferase, induce cell cycle arrest in S and G2/M phase, induce apoptosis, and inhibit metastasis by decreasing CD31, VEGF, IL-8, and mir-21 expression [244–247]. Mechanism of curcumin in CRC as applied to apoptosis is shown in Figure 6. Curcumin has proved to be beneficial in combination with chemotherapy and radiotherapy as well [248, 249]. Synergistic activity of curcumin has also been observed in our study in different sequences and doses with oxaliplatin and cisplatin in four colorectal cell lines (unpublished data).

#### 5.4.3. Rosmarinic Acid and Gingerol

Rosmarinic acid is the main component of Rosemary which at high dose showed antitumour activity applying to* in vitro* and DMH-induced* in vivo* study against CRC [250, 251]. It is thought that MAPK/ERK pathway is linked with the apoptotic mechanism of rosmarinic acid in CRC [252]. Rosmarinic acid has also been reported to possess antimetastatic activity [253].

6-Gingerol is the most important ingredient of ginger showing antiproliferative activity in a dose dependent manner against LoVo cell lines via G2/M cell cycle arrest. Exposure to 6-gingerol induced intracellular ROS and upregulate p53, p27Kip1, and p21Cip1 levels leading to consequent decrease of CDK1, cyclin A, and cyclin B1 [254]. In HCT-115 cell line, its anticancer action was found to be mediated by inhibition of Leukotriene A4 hydrolase [255]. Another study showed that 6-gingerol stimulated apoptosis through upregulation of NAG-1 and G1 cell cycle arrest through downregulation of cyclin D1 that involved protein degradation as well as *β*-catenin, PKC*ε*, and GSK-3*β* pathways [256]. However, 6-gingerol did not show anticancer activity in Colo-205 cell line [257].

#### 5.4.4. Ellagic Acid, Secoisolariciresinol, and Matairesinol

Ellagic acid is a dilactone of hexahydroxydiphenic acid that occurs naturally in berries and nuts such as the raspberry, strawberry, walnut, and pecan. It inhibited growth of Caco-2 cell line, possibly mediated by regulation of matrix metalloproteinases, vascular endothelial growth factor expression, and induction of apoptosis [258]. According to others, the anticancer action is mediated through downregulation of cyclins A and B1 and upregulation of cyclin E, cell cycle arrest in S phase, induction of apoptosis via intrinsic pathway (Fas-independent, caspase 8-independent) via Bcl-XL downregulation with mitochondrial release of cytochrome c into the cytosol, and activation of initiator caspase-9 and effector caspase-3 [54]. Studies on DMH-induced colon carcinogenesis also proved the beneficial effect of ellagic acid and showed the mechanism to be linked with reduced expressions of NF-*κβ*, COX-2, iNOS, TNF-a, and IL-6 as well as inhibition of AKT-phosphoinositide-3 kinase pathway [259, 260]. However, metabolic products urolithins were found to be more potent against CRC compared to ellagic acid itself [261]. Mixed urolithins and ellagic acid inhibited phenotypic and molecular colon cancer stem cell features as well [262].

Secoisolariciresinol and matairesinol are lignans. They constitute one of the major groups of phytoestrogens that have been investigated against CRC but the activity remains controversial. Earlier study with flax seed diet containing secoisolariciresinol and matairesinol showed significant antitumour activity [263]. However,* in vivo* and* in vitro *studies done later did not provide insights into the anticancer potential of secoisolariciresinol and matairesinol [264, 265].

## 6. Chemical Modifications of PP in Nature and Synthetic Analogues

The most important impediment to the successful development of natural PP as clinical therapy against CRC is their low bioavailability. To overcome the problem towards reaching therapeutic concentrations of PP and increasing efficacy, many researchers tried to produce a number of synthetic analogues through structural modifications. Increase in potency* in vitro* and bioavailability* in vivo* has been observed in many studies. Some selected reports concerning chemical modifications of PP applied against CRC are represented in Table 3.

## 7. Combination of Polyphenol with Chemotherapy/Radiotherapy and Other PP

Emerging evidence suggests that a single-agent approach is probably less likely to be very effective in the management cancer. The rationale for recommending a multidrug regimen is to attack cancer cells through multiple targets and diverse mechanisms of actions with reduced toxicity, ultimately leading towards improved clinical outcomes. With that aim, PP have been investigated with chemotherapy and radiotherapy in various cancer models. For example, curcumin given in combination potentiated the cytotoxic effects of doxorubicin, 5-FU, and paclitaxel in prostate cancer cells [266]; enhanced the antitumour activities of cisplatin, doxorubicin, and Taxol in HA22T/VGH hepatic cancer cells, HeLa cells, or CAOV3 and SKOV-3 ovarian cancer cells [267, 268]; sensitized multiple myeloma cells to vincristine and melphalan [269]. Similar evidence is available in literature for resveratrol, EGCG, quercetin, genistein, proanthocyanidin, and daidzein in various types of cancer with different classes of chemotherapeutic drugs [169, 270–273]. A few studies also have been conducted against CRC where it has been observed that PP in combination with other chemotherapeutic drugs produced synergism, for example, curcumin with 5-fluorouracil against HCT-116 and HT-29 cell line, resveratrol metabolites and oxaliplatin in SW-480 and SW-620 cell lines, genistein with 5-fluorouracil in HT-29 cell line [274–277]. Outcomes of some other combination studies already have been mentioned in describing the effect of individual PP. Like chemosensitization, PP also have shown the potential of radiosensitization in various cancers, but investigations of the same effects against CRC is very scarce [278–281]. In one study quercetin has been shown to increase chemoradiosensitivity against colorectal cancer in xenograft mouse model [109].

Plenty of evidence exists in literature regarding the benefits of the effect of one polyphenol combined with another polyphenol against different types of cancer. Combination of resveratrol with black tea polyphenols resulted in a synergistic tumour suppressive response in mouse skin tumour [282]. Resveratrol showed better chemopreventive response when combined with curcumin by maintaining adequate zinc and modulating Cox-2 and p21 level in mouse model of lung cancer [283]. Combination of genistein with resveratrol reduced the most severe grade of prostate cancer in SV40 Tag-targeted probasin promoter rat model [284]. EGCG in combination with curcumin synergistically inhibited oral premalignant epithelial cells [285]. Quercetin and resveratrol in combination with ellagic acid showed synergism against leukaemia [286]. However, studies on the effect of combination of pure polyphenols against CRC are not numerous. Curcumin showed synergistic antitumour effects in combination with resveratrol in one report of colon cancer model in SCMID mice [287]. Combination of epicatechin and EGCG also exhibited synergistic outcome against HT-29 cancer cells. There are few reports in literature related to the beneficial effects of plant extracts or juices that possess mixture of polyphenols against CRC [288, 289].

**Figure 7:**
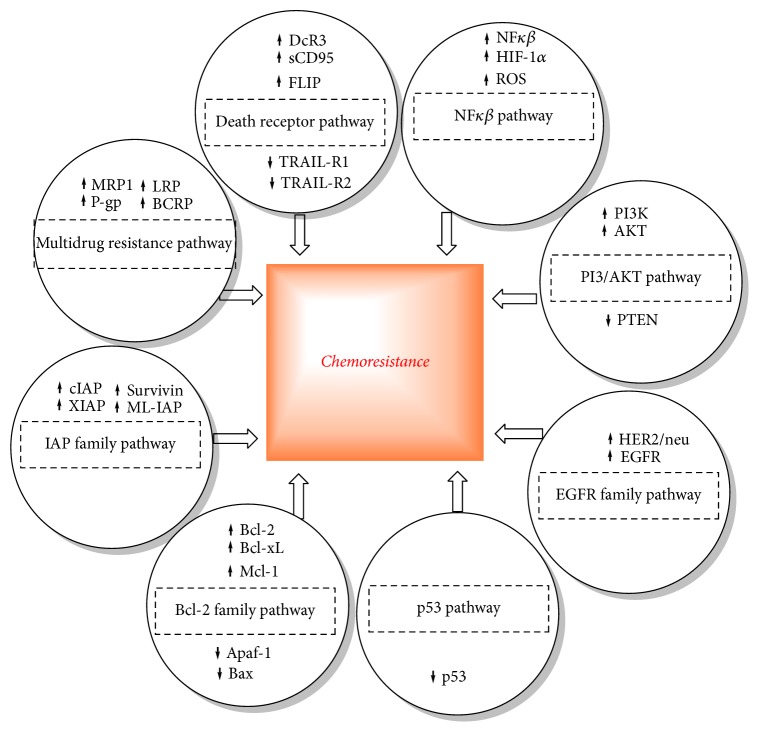
Mechanisms of chemoresistance (adapted from [169]).

Bioprospecting and molecular pharmacology studies have shown that PP can modulate the survival pathways induced by cancer cells, carcinogens, and chemotherapeutics. The possible mechanisms of chemoresistance are shown in Figure 7.

In Section 5, we have considered the molecular mechanisms by which PP would produce anticancer action in CRC. It is thought that PP have the ability to effectively modulate the various mechanisms of chemoresistance. For example, EGCG and quercetin can directly inhibit PI3/AKT pathway, NF*κβ* pathway, EGFR family pathway, and IAP family pathway and increase p53 (Figure 4). Similarly curcumin can inhibit Bcl-2 family pathway, EGFR family pathway, and NF*κβ* pathway (Figure 6).

## 8. Current Status of PP in Clinical Trials Related to CRC

Following the discovery of significant anticancer potential of curcumin, resveratrol, EGCG, and genistein seen in studies related to* in vitro* and* in vivo* rodent model, the compounds entered into clinical trials for efficacy and toxicity study in human model. Many of the reported Phase I, Phase II, and Phase III studies further validated the potential of using PP against CRC. Benamouzig and Uzzan provided a summary of 21 clinical trials conducted by 2016 related to the use of curcumin against CRC in their review [243]. Similarly 17 clinical trials on resveratrol have been reviewed elsewhere [290]. Few other chemotherapeutics and chemoprevention clinical trials conducted on PP have been summarized by Vinod et al. [169].  Table 4 represents the recently completed or ongoing clinical trials on PP against CRC, which have not been covered by others.

## 9. Future Perspective and Directions

From our study we have found that curcumin, resveratrol, quercetin, luteolin, apigenin, EGCG are the most investigated polyphenols against CRC. In terms of* in vitro* cytotoxicity these polyphenols gave average IC_50_ value around 15–60 *μ*M against different colorectal cancer cell lines, which is comparatively larger than clinically used anticancer drugs. Moreover some results from single administration of curcumin or resveratrol produced contradictory evidence against* in vitro* and* in vivo* model data. It would be unwise to be overoptimistic and battle against CRC with a single polyphenol only. Rather the strength of polyphenols can be exploited by combining them with clinically used chemotherapeutic drugs to reduce dose related side effects of chemotherapy and overcoming drug resistance. Therefore, we can search among polyphenols for the ones that give synergistic effect with chemotherapeutic drugs or with other phytochemicals. In our laboratory we have been working with curcumin, resveratrol, EGCG, quercetin, capsaicin, 6-gingerol, genistein in combination with cisplatin and oxaliplatin against different CRC cell lines. In many cases we found synergism (unpublished data) like others [291]. Chemical modifications of natural PP can be continued to improve their activity and bioavailability.

Another area of research that could be explored is related to exploiting ability of PP to interact with stem cells in CRC. Cancer stem cells are multipotent cells that possess self-renewal capacity and high proliferative capacity and lead to metastasis through migration. Although cancer stem cells represent less than 2.5% of the tumour mass, they may be responsible for the resistance to cancer therapies and relapse in CRC [292]. Wnt/*β*-catenin, Hedgehog, and Notch have been identified to play pivotal roles in cancer stem cells self-renewal. Presently researchers are targeting the hallmark stem cell-like properties of tumour cells to overcome cancer. A number of phytochemicals have also been investigated against cancer stem cells in several studies. Curcumin suppressed mammosphere formation along serial passage in breast cancer, and the effect of curcumin on breast cancer stem/progenitor cells was seen to be mediated through its potent inhibitory effect on Wnt/*β*-catenin signalling [293]. Genistein also reduced breast cancer stem cells by inhibiting AKT and increasing PTEN [294]. Likewise, resveratrol inhibited pancreatic cancer stem cell characteristics in human and mouse model by inhibiting EMT [295]. In pancreatic cancer model quercetin decreased ALDH1 activity, induced apoptosis, and reduced the expression of proteins implicated in EMT* in vitro*, while it inhibited stem cell-derived xenografts* in vivo*, reducing the expression of proliferation, stemness, and angiogenesis related genes [296]. However, very little has been studied to modulate the stem cells by PP in CRC.

## 10. Conclusion

As oxidative stress is an inescapable part of aerobic life, it can be said that cancer with its origin in mutations is a disease of living even though it evokes death sentence in many minds. However, as nature creates problems, it also provides solutions. As tumour active polyphenols have been a part of human diet for thousands of years, but without any adverse side effect, it is thought that selected tumour active polyphenols or their derivatives in combination with targeted therapy may provide an affordable means of overcoming drug resistance and reducing side effects in colorectal cancer and indeed in many other cancers.

## Figures and Tables

**Table 1:** Important flavones studied against CRC.

Name	Cell line/animal	Comments	Ref.
Apigenin	SW480, HT-29, and Caco-2	Inhibited colon carcinoma cell growth by inducing a reversible G2/M arrest, associated with inhibited activity of p34cdc2 kinase, reduced accumulation of p34cdc2 and cyclin B1 proteins.	[63]

Apigenin	HCT-8	Suppressed tumour angiogenesis via HIF-1 and VEGF expression.	[64]

Apigenin	HCT-116, SW480, HT-29 and LoVo; APC^Min/+^ mice	Cell death due to apoptosis is mediated by induction of proapoptotic proteins (NAG-1 and p53), cell cycle inhibitor (p21), and kinase pathways.* In vivo *data also supported *in vitro* results.	[65]

Apigenin	HT-29	Cytotoxic activity is related to cell cycle arrest through activation of caspase cascade and stimulation of apoptosis. Synergistic activity observed with 5-FU.	[66]

Apigenin	HT-29 and HRT-18	Inhibited metastasis by upregulating CD26 and degrades CXCL12 by increasing DPPIV activity.	[67]

Apigenin	Xenograft of SW480 cells in nude nice	Suppressed growth of colorectal cancer xenografts via phosphorylation and upregulated FADD expression.	[68]

Apigenin	SW480, DLD-1, and LS174T	Inhibited tumour growth and metastasis both *in vitro* and *in vivo* by upregulating TAGLN, downregulating MMP-9 expression, decreasing phosphorylation of Akt at Ser473 and in particular Thr308.	[69]

Apigenin	Xenograft study using DLD1, HCT-116, HT-29, HCT-8, and SW480	Synergistic effect was observed with ABT-263 and cell death is mediated via inhibition of Mcl-1, AKT, and ERK pathways.	[70]

Apigenin	HCT116	Induced cell death due to apoptosis and autophagy where apoptosis is via decreased expression of cyclin B1, Cdc2, and Cdc25c; increased expression of p53 and p21*CIP1/WAF1*; decreased levels of procaspase-8, -9, and -3.	[71]
	HT-29 and HCT-15	Oxidative stress resulted in senescence and chemotherapeutic effect.	[72]
	SW480 and HCT-15	Suppressed cell proliferation, migration, and invasion via inhibition of the Wnt/*β*-catenin signalling pathway.	[73]
	Sprague Dawley rats	Lowered the number of aberrant crypt foci (ACF) significantly.	[74]

Apigenin, luteolin, baicalein	LoVo and DLD-1	Apigenin had IC_50_ values in LoVo and DLD-1 cells lines at 44.7 *µ*M and 29.6 *µ*M, luteolin at 57.6 and 40.1 respectively. Baicalein has IC_50_ value 51.4 *µ*M in DLD-1 cell line but no significant activity in LoVo cell lines.	[75]

Apigenin, luteolin, tangeretin, nobiletin	Colo 205	After 24-hour exposure, IC_50_ value for apigenin was greater than 100 *µ*M. For luteolin, tangeretin, and nobiletin the values were 47.6 *µ*M, 37.5 *µ*M, and 66.2 *µ*M, respectively.	[76]

Apigenin, baicalein, luteolin, tangeretin, diosmetin	HT-29 and Caco-2	IC_50_ values ranged from 49.4 *µ*M to 203.6 *µ*M in HT-29 cell lines and the trend was baicalein < tangeretin < luteolin < apigenin < diosmetin. For Caco-2 cell lines the trend was baicalein < tangeretin < luteolin < diosmetin < apigenin with values ranging from 56.4 *µ*M to 1115.4 *µ*M.	[77]

Luteolin	HT-29	Downregulated the activation of the PI3K/Akt and ERK1/2 pathways via reduction in IGF-IR signalling which may be one of the mechanisms responsible for the observed apoptosis and cell cycle arrest.	[78]

Luteolin	HT-29, SW480	In HT-29 cells, IC_50_ value was greater than 200 *µ*M but in SW480 cells it is 90 *µ*M.	[79, 80]
	Male Balb/c mice	Inhibited azoxymethane-induced colorectal cancer growth through activation of Nrf2 signalling; altered carbohydrate metabolizing enzymes; decreased expressions of iNOS and COX-2; restored reduced glutathione and protein thiols; decreased lysosomal enzymes, induced apoptosis by modulating Bcl2, Bax, and caspase-3; decreased mucin depleted foci, levels of glycoconjugates; controlled cell proliferation by inhibiting wnt/*β*-catenin/GSK-3*β* pathway. Luteolin also acts as antimetastatic agent by decreasing MMP-9 and MMP-2.	[81–88]
	HCT-15	Induced growth arrest by inhibiting wnt/*β*-catenin/GSK-3*β* signalling pathway, induces apoptosis by caspase-3 mediated manner.	[89]
	HT-29	Induced cell cycle arrest by inhibiting CDK2 and cyclin D1, induces apoptosis by activating caspase-3, -7, and -9.	[90]
	Wistar rats	Decreased the number and volume of 1,2-dimethyl hydrazine induced colon cancer and increased activities of enzymic and nonenzymic antioxidants.	[91, 92]

Pinocembrin	HCT-116, SW480	IC_50_ value in SW480 cell line was 50 *µ*M and <100 *µ*M in HCT-116 cell line. Pinocembrin triggers Bax-dependent mitochondrial apoptosis.	[93]

Tangeretin	HCT-116, HT-29	IC_50_ values were 22 *µ*M and 26 *µ*M, respectively.	[94]
	Colo 205	Induced cell-cycle G1 arrest through inhibiting cyclin-dependent kinases 2 and 4 activities as well as elevating CDK inhibitors p21 and p27.	[76]
	LoVo and multidrug resistant LoVo/Dx	Greater activity was observed against resistant cells more than LoVo cells and gave synergistic effects with doxorubicin by increasing accumulation and sensitizing doxorubicin. It also induced caspase-3 activation and elevated surface phosphatidylserine exposure.	[95]
	HCT-116 and HT-29	*In vitro *and *in vivo *anticancer activity of tangeretin against colorectal cancer was enhanced by emulsion-based delivery system.	[96]

Vitexin-2-O-xyloside	LoVo and Caco-2	Showed IC_50_ values greater than 100 *µ*g/mL in both cell lines but synergistically affected cell growth and apoptosis with raphasatin and (−)-epigallocatechin-3-gallate.	[97]

Nobiletin	F344 rats Sprague Dawley rats	Study on PhIp-induced cancer in F344 rats indicated that nobiletin did significantly reduce the total number of colonic aberrant crypt foci (ACF) compared to the control value.	[74, 98]

Baicalein, wogonin	HT-29 Xenograft assay in nude mouse	IC_50_ values for baicalein and wogonin after 48 h exposure were 100 *µ*M and 150 *µ*M, respectively. *In vivo* data supported the activity of baicalein but wogonin proved to be ineffective. Baicalein induced apoptosis in HT-29 cells via Akt inactivation and in a p53-dependent manner.	[99]

Baicalein	DLD-1 (mutant p53), SW48 (p53 wild-type), and HaCaT	Proteomic study proved that baicalein upregulated the expression of PRDX6, which attenuates the generation of ROS and inhibits the growth of CRC cells.	[100]

**Table 2:** Important flavonols studied against CRC.

Name	Cell line/animal	Comments	Ref.
Quercetin	SW480 and HT-29	Inhibited cell growth and induced apoptosis via downregulation of ErbB2/ErbB3 signalling and the Akt pathway.	[101]
	Wistar rats	During DMH induced colon cancer assay, quercetin inhibited intestinal crypt cell proliferation *in vivo*, but the effect diminished as the level of dietary exposure increased.	[33]
	SW480	Inhibited *β*-catenin/TCF signalling.	[102]
	CACO-2 and HT-29	Had IC_50_ values in the range 30–40 *µ*M.	[103]
	CO115 and HCT15	Produced synergistic effect in combination with 5-FU by increasing apoptosis via modulating p53.	[104]
	HT-29 xenografts in male nude mice	Induced apoptosis via AMPK activation and p53-dependent apoptotic cell death. Another study using HT29 cell line indicated that quercetin inhibited phosphorylation of EGFR and the ErbB2 receptor.	[105, 106]
	SW480	Antitumour action in SW480 colon cancer cells is related to the inhibition of expression of cyclin D1 and survivin through Wnt/*β*-catenin signalling pathway.	[107]
	HT-29	Resveratrol and quercetin in combination showed anticancer activity in colon cancer cells and repressed oncogenic microRNA-27a.	[108]
	HT-29 xenografts in female nude mice	Quercetin and trans-pterostilbene in combination facilitated elimination of colorectal cancer by chemoradiotherapy through a Bcl-2- and superoxide dismutase 2-dependent mechanism.	[109]
	CF1 mice, F344 rats, Wistar rats	Azoxymethane and dimethylhydrazine induced colon cancer study showed reduction of aberrant crypt foci and focal areas of dysplasia.	[110–115]
	APC^Min/+^ mouse	Quercetin reduced polyp number and size distribution, which might be due to a reduction in macrophage infiltration.	[116]

Quercetin, myricetin, fisetin, galangin, chrysin, morin	LoVo and DLD-1	In LoVo cell lines the trend of IC_50_ values was fisetin < myricetin < quercetin < galangin < chrysin, whereas in DLD-1 cell line it was fisetin < myricetin < galangin < quercetin < chrysin. No significant antitumour effect was observed for Morin.	[75]

Quercetin, chrysin, kaempferol	SW480	Quercetin, chrysin, and kaempferol gave IC_50_ values of 85, 165, and 100 *µ*M, respectively.	[80]

Myricetin	HCT-115, Colo-205	Myricetin induced cell death of human HCT-115 cells via Bax/Bcl2-dependent pathway. It inhibited matrix metalloproteinase 2 protein expression and enzyme activity in Colo-205 cells.	[117, 118]

Rutin	SW480, Nude mice	Rutin gave IC_50_ value of 125 *µ*M and exerted *in vivo* antitumor and antiangiogenic activities.	[119]
	HT-29	Induced mitochondrial apoptosis through a caspase-dependent mechanism.	[120]
	CF1 –female mice	Inhibited azoxymethane-induced colonic neoplasia.	[113]

Chrysin	HT-29	Had IC_50_ value of 3.1 *µ*M.	[121]
	HCT-116	Chrysin sensitized tumour necrosis factor-*α*-induced apoptosis in human tumor cells via suppression of nuclear factor-kappaB.	[122]
	HCT-116	Promoted tumour necrosis factor- (TNF-) related apoptosis-inducing ligand (TRAIL) induced apoptosis.	[123]
	SW480	Chrysin caused cell-cycle arrest at the G2/M phase in a dose-dependent manner.	[80]
	HCT116, DLD1 and SW837	Aryl hydrocarbon receptor was required for the chrysin induced apoptosis and the upregulation of *TNF-α* and *-β*gene expression and consequent activation of the TNF-mediated transcriptional pathway.	[124]
	Caco-2	Blocked topotecan-induced apoptosis in spite of inhibition of ABC-transporters.	[125]

Kaempferol	SW480	Sensitized TRAIL-induced apoptosis.	[126]
	HCT-116	The IC_50_ of kaempferol was 53.6 *µ*M in HCT116 (p53+/+) cells and 112.7 *µ*M in HCT116 (p53−/−) cells. It induced via ataxia-telangiectasia mutated-p53 pathway with the involvement of p53 up-regulated modulator of apoptosis.	[127]
	HT-29	Kaempferol increased chromatin condensation, DNA fragmentation, and the number of early apoptotic cells in a dose-dependent manner. Kaempferol increased the levels of cleaved caspase-9, caspase-3, and caspase-7 as well as those of cleaved poly (ADP-ribose) polymerase. Moreover, it increased mitochondrial membrane permeability and cytosolic cytochrome c concentrations.	[128]

Isorhamnetin	HT-29, FVB/N mice	Chemoprotective effects of isorhamnetin were linked to its inhibition of oncogenic Src activity and consequential loss of nuclear *β*-catenin, activities that were dependent on CSK expression.	[129]
	HCT-116, SW480 and HT-29	IC_50_ values for isorhamnetin in HCT-116, SW480, and HT-29 cell lines were 54.87, 56.24, and 43.85 *µ*M, respectively. The mechanism of cell death was linked with PI3KAktmTOR pathway.	[130]

Fisetin	HT-29	Fisetin inhibited cyclin-dependent kinases leading to cell cycle arrest.	[131]
	HT-29	Enhanced radiosensitivity of p53-mutant HT-29 human colorectal cancer cells.	[132]
	HCT-116, HT-29	IC_50_ values for fisetin in HCT-116 and HT-29 cell lines were 132.2 and 57.7 *µ*M after 72 h, respectively. The mechanism was induction of apoptosis by inhibition of COX2 and Wnt/EGFR/NF-kB-signalling pathways.	[133]
	HCT-116	Securin depletion sensitizes human colon cancer cells to fisetin-induced apoptosis.	[134]

Galangin	HCT-15, HT-29	Induced cell death via mitochondrial dysfunction and caspase-dependent pathway.	[135]

Morin	HCT-116	Had IC_50_ value less than 350 *µ*g/mL after 48 h and induced apoptosis by modulation of Bcl-2 family members and Fas receptor.	[136]

**Table 3:** Important synthetic polyphenols studied against CRC.

Parent PP	Synthetic analogue	Activity	Ref.
Pterostilbene	3′-Hydroxy-pterostilbene	In terms of IC_50_ values, synthetic analogue found to be more sensitive against 3 CRC cell lines. *In vivo* study also proved its greater activity.	[137]

Resveratrol	3, 5, 4′-Trimethoxystilbene, 3, 3′, 4, 5′-tetramethoxystilbene	Inhibited HT-29 cell growth.	[138]
	Digalloyl resveratrol	Inhibited HT-29 cell growth more effectively than gallic acid and resveratrol.	[139]

Flavone	3′, 4′, 5′, 5, 7-Pentamethoxyflavone	More active compared to tricin and apigenin in APC^Min/+^ mice model.	[140]

Curcumin	Dimethoxycurcumin	In HCT-116 cell lines, dimethoxycurcumin is more potent in terms of ability to kill cancer cells by apoptosis, less extensively metabolized in microsomal systems, and more stable *in vivo* compared to curcumin.	[141]
	Curcumin difluorinated	At higher concentration synthetic analogue showed greater potency than curcumin in HCT-116 cells.	[142]
	EF31 and UBS109	Both analogues showed significant antitumour activity in colorectal xenograft model possibly via inhibition of NF-*κ*B and cell cycle progression at G0/G1 phase.	[143]
	GO-Y030, FLLL-11, and FLLL-12	All of the analogues exhibited 4 to 20 times greater activity than curcumin against SW480, HT-29, and HCT116 cell lines but with minimal toxicity against normal cell line.	[144]

EGCG	Peracetylated EGCG	Administration of the synthetic analogue was more effective than EGCG in preventing the shortening of colon length and the formation of aberrant crypt foci and lymphoid nodules in mouse.	[145]

Procyanidin dimer	[3-O-Galloyl]-(−)-epicatechin-(4*β*,8)-(+)catechin-3-O-gallate	Compared to parent compound synthetic analogue showed increased cytotoxicity against twelve different cell lines including two colorectal cell lines.	[146]

Catechin and/or epicatechin	(2R, 3S)-3′, 4′, 5,7- tetrahydroxyflavone-3-yl decanoate, (2R, 3S)-3′, 4′, 5,7-tetrahydroxyflavone-3-yl octadecanoate	Both of them exerted greater cytotoxicity in HCT-116 cells than catechin.	[147, 148]

Genistein	4′-O-(3,4-Di-O-acetyl-*α*-L-arabino–hexopyranosyl) genistein, 7-O-(2,3,4,6-tetra-O-acetyl-*β*-D-galactopyranosyl)-(1→4)-(6-O-acetyl-hex-2-ene-*α*-D-erythro-pyranosyl)genistein, 7-O-(2,3,5-tri-O-benzyl-*β*-D-arabinofuranosyl)genistein and 7-O-(4,6-di-O-acetyl-hex-2-ene-*α*-D-erythropyranosyl)genistein	The derivatives showed greater cytostatic and cytotoxic effect than genistein in Colo-205 cell lines.	[149]

Epicatechin	3-O-(3,4,5-trimethoxybenzoyl)-(−)-epicatechin	Synthetic analogue showed IC_50_ values at 33 *µ*M against Caco-2 cell lines and greater activity compared to epicatechin.	[150]

Naringenin	6-C-(E-phenylethenyl)- naringenin	6-C-(E-phenylethenyl)-naringenin suppressed CRC without any toxicity by inhibiting cyclooxygenase-1.	[151]
	5-Hydroxy-2-(4-hydroxyphenyl)-4-oxochroman-7-yl thiophene-2-carboxylate, 5-hydroxy-2-(4-hydroxyphenyl)-4-oxochroman-7-yl 2-methylbenzoate, 5-hydroxy-2-(4-hydroxyphenyl)-4-oxochroman-7-yl isobutyrate, 7-(allyloxy)-5-hydroxy-2-(4-hydroxyphenyl) chroman-4-one and 5-hydroxy-2-(4-hydroxyphenyl)-4-oxochroman-7-yl phenyl carbonate	All of the derivatives gave lower IC_50_ values compared to naringenin in HCT-116 cell lines.	[152]

Chrysin	5,7-dimethoxy-8-iodochrysin, 8-bromo-5-hydroxy-7-methoxychrysin and 5,7-Dihydroxy-8-nitrochrysin	These three derivatives among twelve prepared analogues showed prominent activity against CRC compared to chrysin.	[121]

Nobiletin or/and tangeretin	5-hydroxy-6,7,8,3′,4′-pentamethoxyflavone, 5-hydroxy-3,6,7,8,3′,4′-hexamethoxyflavone, and 5-hydroxy-6,7,8,4′-tetramethoxyflavone.	All synthetic analogues showed lower IC_50_ values than nobiletin and tangeretin.	[94]

**Table 4:** Recent clinical trials on PP against CRC.

Polyphenol	Study description (patients)	Institution and status
Curcumin	A Phase II, randomized, double blind, placebo controlled trial for the effectiveness of holistic turmeric supplementation on polyp burden among patients with FAP (40)	Tel Aviv Sourasky Medical Center, Israel; started February 2017

Curcumin	Early Phase I, curcumin in combination with 5-FU in chemoresistant metastatic colorectal cancer (14)	Baylor Research Institute, USA; started March 2016

Curcumin	Randomized Phase II trial studies in treating patients with FAP (44)	Johns Hopkins University USA; completed in 2017 but results have not been published yet

Curcumin	Phase I, pharmacokinetic trial of curcuminoids (24)	University of Michigan Cancer Center, USA; completed but no publication

Curcumin	Phase I, microarray analysis to identify genes that are modified by curcumin that could be used as biomarkers (40)	University of North Carolina, USA; completed but no publication

EGCG	Phase I, chemopreventive effects in patients with curative resections (50)	The University of Texas Health Science Center at San Antonio, USA; started January 2017 and recruiting

EGCG	Green tea extracts for the prevention of colorectal adenomas and colorectal cancer (176)	Seoul National University Hospital, South Korea; completed and found favourable outcome for the chemoprevention [153]

Genistein	Phase I/II, incorporation of genistein in FOLFOX treatment regimen against metastatic CRC (13)	Sofya Pintova, Icahn School of Medicine at Mount Sinai in collaboration with DSM Nutritional Products, Inc., USA; completed January 2017 but result has not been published yet

## Abbreviations

ABCB1: ATP-binding cassette subfamily B member 1
AKT: Protein kinase B
AMPK: 5′AMP-activated kinase
AP-1: Activator protein-1
Apaf-1: Apoptotic protease activating factor 1
APC: Activated protein C
Bax: Bcl-2-associated X protein
Bcl-2: B-cell lymphoma 2
BCRP: Breast cancer resistance protein
CASP3: Caspase-3
Cdc2: Cell division cycle protein 2
CDK4: Cyclin-dependent kinase 4
c-Fos: Protooncogene
CHOP/GADD 153: Homologous protein/growth arrest- and DNA damage-inducible gene 153
cIAP: Cellular inhibitor of apoptosis protein
Cox-2: Cyclooxygenase-2
CRC: Colorectal cancer
CTGF: Connective tissue growth factor
Cyt-c: Cytochrome complex
DcR3: Decoy receptor 3
DR5: Death receptor 5
ECM: Extracellular matrix
EGFR: Epidermal growth factor receptor
EMT: Epithelial-mesenchymal transition
ERK: Extracellular signal-regulated kinases
FAP: Familial adenomatous polyposis
GADD153: Growth arrest- and DNA damage-inducible gene 153
HIF-1: Hypoxia-inducible factor-1
Hsp70: Heat shock protein 70
IAP: Inhibitor of apoptosis
ICAMs: Intercellular cell-adhesion molecule-1
IGF-1R: Insulin-like growth factor 1 (IGF-1) receptor
IKK: I kappa B kinase
IL-8: Interleukin 8
ILs: Interleukins
INOS: Inducible nitric oxide synthase
IRS-1: Insulin receptor substrate 1
IRS-2: Insulin receptor substrate 2
JAK: Janus kinase
JNK: C-Jun N-terminal kinases
PGE2: Prostaglandin E2
KRAS: Kirsten rat sarcoma
LOX: Liquid oxygen
LRP: Lipoprotein receptor-related protein
MALAT1: Metastasis associated lung adenocarcinoma transcript 1
MAPK: Mitogen-activated protein kinase
MEK: Mitogen-activated protein kinase
miR-34a: MicroRNA 34a
MMPs: Matrix metalloproteinases
MRP1: Multidrug resistance-associated protein 1
mTOR: Mammalian target of rapamycin
Myc: Myelocytomatosis
NADPH: Nicotinamide adenine dinucleotide phosphate
NF-k*β*: Nuclear factor kappa-light-chain-enhancer of activated B cells
P-450: Cytochromes P450
P53: Phosphoprotein p53
PARP: Poly (ADP-ribose) polymerase
PDCD4: Programmed cell death protein 4
P-gp: P-glycoprotein 1
PI3K: Phosphoinositide 3-kinase
PP: Polyphenols
PTEN: Phosphatase and tensin homolog
RECK: Reversion-inducing-cysteine-rich protein with kazal motifs
ROS: Reactive Oxygen Species
SIRT1: Sirtuin 1
TCF: Transcription factor
TGF*β*: Transforming growth factor beta
TGF*β*R1/2: Transforming growth factor beta receptor I
TIMP3: Metalloproteinase inhibitor 3
TNF-*α*: Tumour necrosis factor alpha
TRAIL-R1: Tumour necrosis factor-related apoptosis-inducing ligand-receptor 1
TRAIL-R2: Tumour necrosis factor-related apoptosis-inducing ligand-receptor 2
TSP1: Thrombospondin 1
uPAR: Urokinase receptor
xIAP: X-linked inhibitor of apoptosis protein
ZEB1/2: Zinc-finger E-box-binding homeobox 1 and 2.
